# Dr. Walker’s Paper on Hernia

**Published:** 1887-10

**Authors:** 


### DR. WALKER'S PAPER ON HERNIA.

Our esteemed friend and corresdondent, Dr. W. W. Walker, of Schulenburg, Texas, to whom we are indebted for the valuable paper on Hernia which appeared in our last issue. we regret to learn, took some exceptions to the editorial remarks which accompanied it, and felt that we had questioned the veracity of his statement. Nothing could have been farther from our intentions, and so the Doctor was promptly assured.

The fact that Dr. Walker has, in seventeen years practice met with, and successfully treated some sixty cases of strangulated inguinal hernia, living, as he has done, in the country, is so unusual an experience, that lest any one, not knowing Dr. Walker's high character and standing as a gentleman and a physician, might be disposed to doubt the correctness of the report, we will say that altho' his practice is a county practice, it extends over a large area of the richest and most densely populated portion of Texas, a section settled thickly, by Germans principally. These are a hard laboring class, and being engaged principally in farming, have much lifting and other labor to do, which is calculated to produce rupture. They are gregarious, and their settlement is like a village,—the houses being within a stone's throw of each other, often, and usually, not over a mile apart. It is estimated to contain from twenty-five to thirty thousand inhabitants. Besides this, Dr. Walker, being well known, and very popular with his professional brethren, does a large consulting practice, and it is, under these peculiar and advantageous circumstances, no wonder that he has had such an extensive experience in manipulating hernia. His method is so simple, and so successful in his hands, that we again suggest that our readers, especially younger practitioners, make a note of it.

